# A Flexible Triboelectric Nanogenerator Based on Multilayer MXene/Cellulose Nanofibril Composite Film for Patterned Electroluminescence Display

**DOI:** 10.3390/ma15196770

**Published:** 2022-09-29

**Authors:** Zhaoyang Sun, Huamin Chen, Mingqiang Wu, Wei Yang, Jiang Zhao, Zefeng Wang, Shujun Guo, Huining Wang, Weiguo Wang, Jun Wang

**Affiliations:** 1 School of Materials Science and Engineering, Fujian University of Technology, Fuzhou 350118, China; 2 College of Materials and Chemical Engineering, Minjiang University, Fuzhou 350108, China; 3 Fujian Key Laboratory of Functional Marine Sensing Materials, College of Materials and Chemical Engineering, Minjiang University, Fuzhou 350108, China; 4 Faculty of Science and Engineering, The University of Nottingham Ningbo China, Ningbo 315104, China

**Keywords:** triboelectric nanogenerator, MXene, cellulose nanofibril, flexible electronic, electroluminescence

## Abstract

The flexible self-powered display system integrating a flexible triboelectric nanogenerator (TENG) and flexible alternating current electroluminescence (ACEL) has attracted increasing attention for its promising potential in human–machine interaction applications. In this work, a performance-enhanced MXene/cellulose nanofibril (CNF)/MXene-based TENG (MCM-TENG) is reported for powering a flexible patterned ACEL device in order to realize self-powered display. The MCM multilayer composite film was self-assembled through the layer-by-layer method. The MCM film concurrently acted as a triboelectric layer and electrode layer due to its high conductivity and strength. Moreover, the effect of CNF concentration and number of layers on the output performance of TENG was investigated. It was found that the MCM-TENG realized the optimum output performance. Finally, a flexible self-powered display device was realized by integrating the flexible TENG and ACEL. The MCM-TENG with an output voltage of ≈90 V at a frequency of 2 Hz was found to be efficient enough to power the ACEL device. Therefore, the as-fabricated flexible TENG demonstrates a promising potential in terms of self-powered displays and human–machine interaction.

## 1. Introduction

The rapid development of flexible electronics and electronic skin is providing great convenience to our lifestyle for its application in augmented reality [1,2,3], human–machine interaction [4,5,6], and biomedical applications [7,8]. Moreover, the flexible displays or visualized electronics have become an indispensable interactive medium in view of their efficient and direct information transfer [9,10,11]. Currently, the flexible displays mainly focus on the electroluminescence (EL) [12,13,14], photoluminescence [15,16,17], and triboluminescence [18,19,20]. Among these, the EL device, especially the alternating current electroluminescence (ACEL), stands out on account of its quick response, high brightness, excellent deformability, and easy fabrication [21,22]. Flexible ACEL has been widely used in flexible displays that can be mounted on the human body, and as a flexible sensing platform to sense epidermal information [23,24,25]. However, the inherent high AC driven voltage of ACEL imposes energy supply barriers for applications in flexible systems.

A triboelectric nanogenerator (TENG), based on the Maxwell’s displacement current [26,27], can effectively convert random mechanical energy such as human movement [28,29] and environmental energy [30,31] into electric energy. The TENG possesses excellent electric performance and mechanical properties, including various working modes [32,33,34,35], high output voltage [36,37], high energy conversion efficiency [38,39], and flexibility and biocompatibility [40,41]. Moreover, many strategies including material optimization [42,43], structure modification [44,45], and charge injection [46] have been reported to greatly improve the output performance of TENG. Among them, electrode material is one of the most important factors. Transition metal carbides and nitrides (MXene) are promising candidates for flexible electrodes due to their high conductivity and electronegative surfaces [47,48]. However, most of the MXene material is not directly in contact with the triboelectric layer of MXene-based TENG due to its poor mechanical strength.

These advantages make TENG an indispensable part in the flexible self-powered systems. More importantly, due to the intrinsic high output voltage up to thousands of volts and AC output, the TENG is naturally suitable to power a flexible ACEL device. The self-powered display system integrating flexible TENG and the ACEL has attracted widespread concern for its promising potential in flexible and wearable applications [49,50,51,52,53]. Until now, flexible self-powered ACEL is undergoing a rapid development in reducing the driving voltage and increasing the output voltage.

Herein, we propose a flexible TENG that is based on multilayer MXene/cellulose nanofibrils (CNFs)/MXene composite electrode for powering flexible ACEL. Firstly, the MXene/CNFs/MXene (MCM) composite films were self-assembled through layer-by-layer vacuum filtration. The MCM thin films exhibit high conductivity and strength, which is suitable for flexible electrodes. This is because of the introduction of the CNF layer, which provides the support scaffold for the MCM film. Furthermore, various flexible TENGs based on pure MXene film, MXene/CNFs film, and MCM film were constructed to figure out the effect of CNFs on the output performance of TENG. It was demonstrated that the TENG based on the MCM composite electrode realized the optimum output performance. Finally, the flexible TENG was utilized to drive the flexible ACEL for realizing flexible self-powered display. Consequently, the flexible TENG demonstrated promising potential in self-powered displays and human–machine interaction.

## 2. Materials and Methods

### 2.1. Preparation of Composite Electrode

The multi-layer Ti_3_C_2_T_x_ powder (particle size: 0.2–10 μm) was purchased from Jilin 11 Technology Co., Ltd. (Changchun, China) Then, TMAOH (Aladdin, AR, Shanghai, China) was used to delaminate multi-layer Ti_3_C_2_T_x_. A total of 2 g of multi-layer Ti_3_C_2_T_x_ was suspended in a mixture of deionized water (40 mL) and TMAOH (25% aqueous solution, 2 mL). It was treated by a sonication process for 3 h to obtain a few-layer Ti_3_C_2_T_x_ colloidal solution.

The CNFs (10–20 nm in diameter and 20–1000 nm in length) were purchased from North Century (Xuzhou, China) Cellulose Material Co., Ltd. The MXene thin film was self-assembled via vacuum-assisted filtration. Moreover, the multi-layer composite films such as MXene/CNFs and MCM films were fabricated through a layer-by-layer method. Finally, the as-prepared thin films were cured at 50 °C for 10 min to release the strain; thus, a flat composite electrode was obtained.

### 2.2. Construction of Flexible MXene/CNF-Based TENG

First, polydimethylsiloxane (PDMS, weight ratio of A:B = 20:1) thin film with a thickness of 0.7 mm was prepared through the spin-coating method. Then, the PDMS film was sputter-coated with a layer of Ag via vacuum sputter coater (DP650, Paris, France), followed by an encapsulation layer of PDMS. Thus, a sandwich-like structure was obtained, which can protect the electrode layer from large strain. The bottom electrode was fabricated using the aforementioned method. These two films were connected with an air gap of 5 mm to form the flexible TENG.

### 2.3. Characterization

The surface morphologies and elemental analysis were characterized by a field-emission scanning electron microscope (SEM, SU8010, Hitachi, Tokyo, China) and a 3D scanning microscope (VK-X200 series). The X-ray diffraction (XRD) pattern was acquired by the diffractometer (MiniFlex600, Rigaku, Tokyo, China). A linear motor control system (R-LP3, Beijing, China) was used to adjust the compressive forces and working frequencies. The resistance was measured by a digit multimeter (DMM 6500), and the output electric performance of TENG was measured by an electrometer (Keithley 6514).

## 3. Results and Discussion

**Figure 1:**
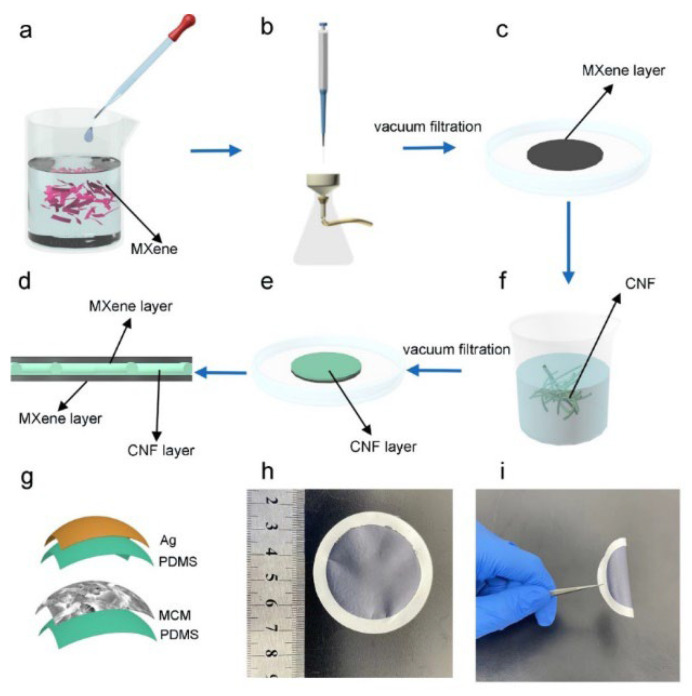
Schematic illustration of a multi-layer MXene composite film and a MXene-based TENG. (**a**) The few-layer MXene solution. (**b**) The self-assembled process via vacuum filtration. (**c**) The MXene thin film. (**d**) The CNF solution. (**e**) A CNF layer was formed on the MXene film. (**f**) The MCM multilayer. (**g**) Schematic structure of the MCM-TENG. (**h**) Photograph of the MCM composite electrode. (**i**) High flexibility of the flexible electrode.

The schematic illustration of the fabrication process of the multi-layer MCM electrode is shown in Figure 1a–f. The few-layer MXene solution was realized by delaminating the multi-layer MXene powder (Figure 1a). Then, the MCM film was fabricated via vacuum-assisted filtration in the following order: MXene-CNF-MXene (Figure 1b–f). Subsequently, a sandwich-like multi-layer electrode was obtained after drying the multi-layer film. The MXene film and the MXene/CNF film were fabricated using the similar method. Figure 1g schematically illustrates the structure of flexible TENG. The top Ag electrode was clamped between the triboelectric layer PDMS and the encapsulation layer. The MCM acted as the bottom electrode. Moreover, the two flexible films made the TENG flexible. The photographs of the MCM thin film are exhibited in Figure 1h. The diameter of the MCM film was 4 cm, and the thickness was 15 μm. In addition, the flexibility of MCM is displayed in Figure 1i. It retained high conductivity under the bending state.

**Figure 2:**
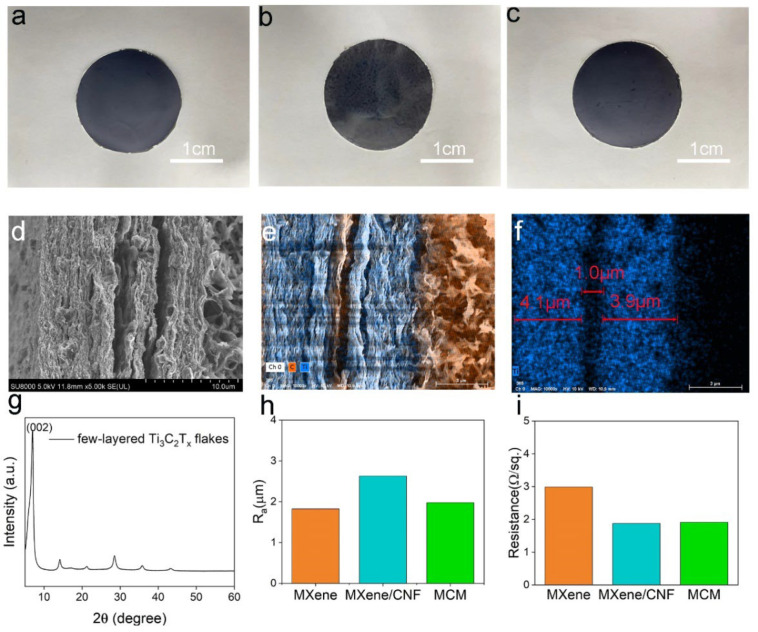
Characteristics of the MCM film. (**a**–**c**) Photographs of MXene, MXene/CNF, and MCM films. (**d**) SEM image of the lateral section of MCM film. (**e**) The energy-dispersive spectrometer of MCM film. (**f**) The Ti element distribution in the MCM film. (**g**) The XRD pattern of few-layered MXene. (**h**) The surface roughness of MXene, MXene/CNF, and MCM films. (**i**) The sheet resistances of MXene, MXene/CNF, and MCM films.

The morphology and electrical properties were characterized in Figure 2. The optical photos of MXene, MXene/CNF, and MCM films are displayed in Figure 2a–c. The fabricated MXene and MCM films showed a smooth surface, while the MXene/CNF film had a relatively rough surface due to the CNF on the surface. To demonstrate the successful fabrication of layered MCM film, the cross-sectional SEM is shown in Figure 2d. It was obvious that the MCM films can be divided into three layers. Furthermore, the element analysis in Figure 2e,f clearly confirmed the layered structure. The Ti element represented the MXene film, and the C element represented the CNF film, as shown in Figure S1 in the Supplementary Materials. The thickness of the MXene and CNF films were indicated to be 4 and 1 μm, respectively. In addition, the XRD pattern with a characteristic peak at 6.2° in Figure 2g can prove the successful removal of the Al component. The other peaks demonstrated that the as-prepared MXene was a few-layer structure. Moreover, the surface morphology was expressed quantitatively by surface roughness, as shown in Figure 2h. The MXene and MCM film exhibited relatively low surface roughness levels of 1.8 μm and 1.9 μm, respectively. Conversely, the MXene/CNF film showed a relatively high surface roughness of 2.6 μm. Theoretically, the higher surface roughness can result in higher output performance of TENG. The electrical property of these films are compared in Figure 2i. The Mxene, Mxene/CNF, and MCM films all achieved small sheet resistance with 3 Ω/sq, 2 Ω/sq, and 2 Ω/sq, respectively. They showed excellent conductivity, which is suitable for a triboelectric electrode.

**Figure 3:**
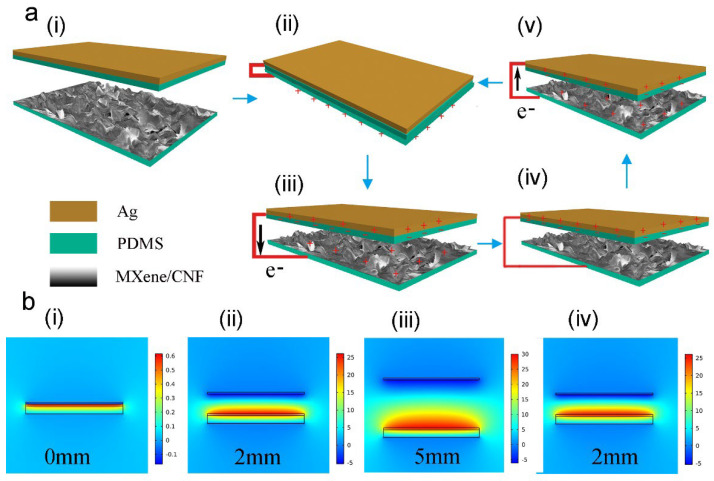
Working mechanism of the MCM-TENG. (**a**) The charge distribution and charge transfer in the contact–separation mode. (**b**) The potential difference between the two electrodes in the contact–separation mode.

A clear understanding of the working mechanism of TENG can help us to improve its output performance. The charge distribution and electron transfer are illustrated in Figure 3a. In the initial state, the two triboelectric layers (MCM and PDMS film) were not in contact, and there were no triboelectric charges (i). Under compressive force, the MCM film and PDMS film were in full contact, leaving the triboelectric charge distributed on the surface (ii). As for releasing the compressive force, the potential difference drove the electron flow from the top electrode to the bottom electrode (iii). When the TENG returned to the initial state, the distance between the MCM film and the PDMS film reached the maximum value (iv). Next, when the MCM film approached the top electrode, the potential difference drove the electron transfer in the opposite direction (v). Finally, when the two triboelectric layers were in contact again, the opposite triboelectric charge was recombined. Moreover, there was no potential difference between the two electrodes. The cyclic contact and separation will result in AC output. Figure 3a schematically illustrates the short-circuit current of TENG, and the finite element analysis of the open-circuit voltage of TENG was conducted, as shown in Figure 3b. In the fully contact state (d = 0 mm), there was nearly no potential difference between the two electrodes (i). When the distance increased to 2 mm, the potential difference increased to ≈30 V (ii). As the distance reached the maximum value, the potential difference also reached the maximum value of ≈35 V (iii). When the distance decreased to 2 mm again, the potential difference decreased to ≈30 V (iv). The open-circuit voltage was proportional to the distance. Thus, the measured maximum voltage will occur when two triboelectric layers are fully separated. Conversely, the measured maximum current occurs between fully contact and the initial state. In addition, the switching polarity test of the TENG was conducted, as shown in Figure S2 in the Supplementary Materials.

**Figure 4:**
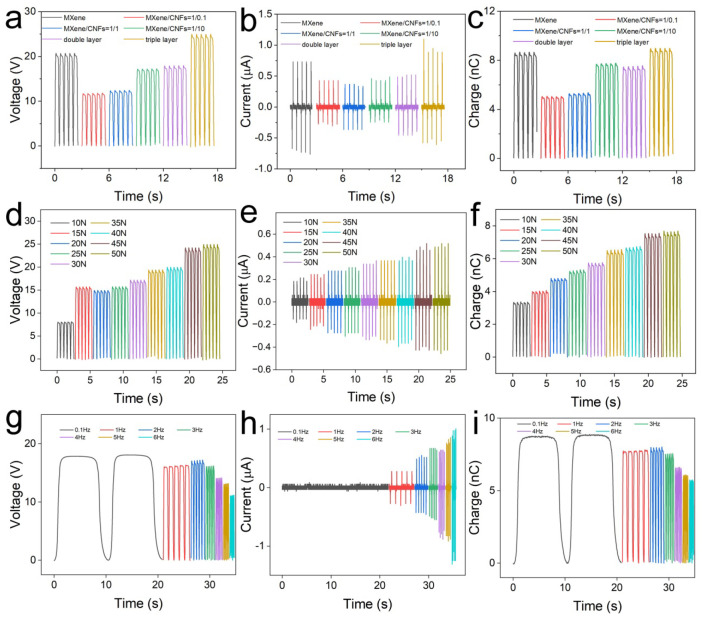
Output electrical performance of TENG. (**a**) Open-circuit voltage, (**b**) output current, and (**c**) transferred charges of TENG based on various bottom electrodes. The relationship between (**d**) open-circuit voltage, (**e**) output current, (**f**) transferred charges of various TENG, and the compressive force. The effect of working frequency on the (**g**) open-circuit voltage, (**h**) output current, and (**i**) transferred charges.

A series of TENGs based on MXene film, MXene/CNF film (with different CNF concentrations), MXene-CNF film, and MCM film were fabricated to figure out the CNF concentration and the number of layers on the output performance of TENG. For convenience, the single MXene/CNF layer with various CNF concentrations (MXene/CNF = 0.1, 1, and 10) were represented as M_0.1_, M_1_, and M_10_, respectively. In addition, the double layer MXene-CNF layer was marked as MC. The output voltage of TENG based on various electrodes is compared in Figure 4a. The single-layer electrode-based TENG showed output voltages of 20.7 V (MXene), 11.8 V (M_10_), 12.4 V (M_1_), and 17.2 V (M_0.1_). Obviously, the MXene-based TENG obtained the relatively high output voltage. This was due to the lower conductivity as the CNF concentration increased. Moreover, the TENG based on MC film and MCM film exhibited output voltages of 17.9 V and 25.0 V, respectively. For the MC-based TENG, the MXene acted as the bottom electrode, and CNF acted as a triboelectric layer. The triboelectric effect between the PDMS and MXene was stronger than that between PDMS and CNF. Moreover, for MCM-based TENG, the CNF between the two MXene films can effectively block the combination of triboelectric charge and induced charge. The output current and transferred charges exhibited a similar trend. As shown in Figure 4b,c, the MCM-based TENG achieved the highest output performance with an output current of 0.95 μA and transferred charges of 9.0 nC.

Furthermore, we investigated the effect of experimental parameters such as external force and working frequency on the output performance in detail. The output performance of MCM-based TENG was measured at a frequency of 1 Hz. As shown in Figure 4d, the output voltage increased from 8.0 to 24.9 V as the external force increased from 5 to 50 N. The output voltage was enhanced threefold. The relationship between the output current, transferred charges, and the external force is shown in Figure 4e,f. The output current was enhanced from 0.2 to 0.5 μA, and the transferred charges were enhanced from 3.4 to 7.7 nC as external force increased from 5 to 50 N. The higher external force represents more input mechanical energy, which results in higher output performance. However, it should be noted that the energy conversion efficiency gradually decreased as the external force increased. The influence of working frequency on the output performance is displayed in Figure 4g–i. The output voltage was slightly decreased from 17.8 V (0.1 Hz) to 16.1 V (3 Hz), and sharply decreased to 11.1 V as the frequency increased from 3 to 6 Hz. This was due to inadequate contact between two triboelectric layers, leading to less triboelectric charge. The transferred charge behaved similar to the relationship between transferred charge and the frequency. On the contrary, the output current increased rapidly from 0.1 to 1.0 μA as the frequency increased from 0.1 to 6 Hz. This enhancement can be attributed to the higher electron flow.

**Figure 5:**
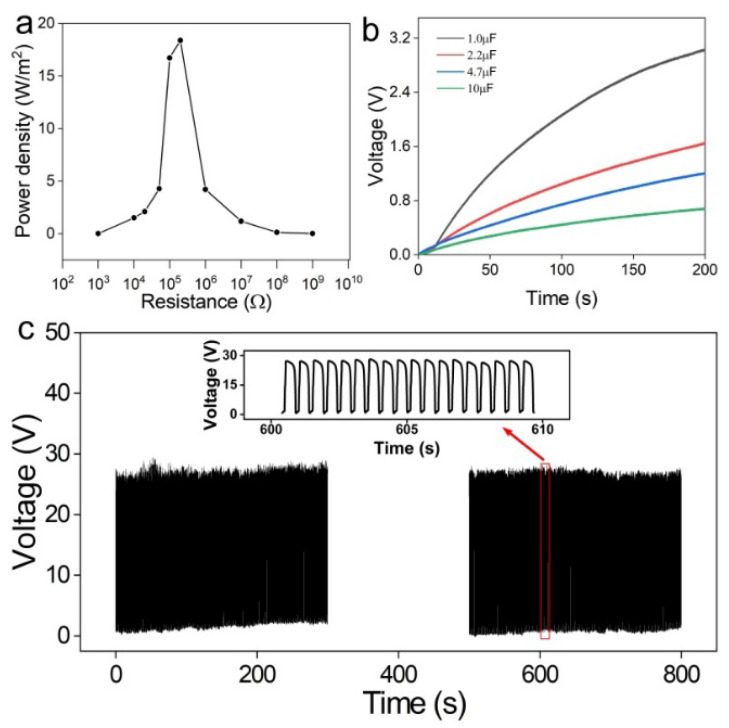
Charging ability of the MCM-TENG. (**a**) The relationship between power density and load resistance. (**b**) Charging curve of different capacitors (1 μF, 2.2 μF, 4.7 μF, and 10 μF). (**c**) The stability test of about 2000 cycles.

Powering capacity is another parameter used to assess the electrical performance of TENG. The output power density of MCM-TENG is shown in Figure 5a. The MCM-TENG was working under a force of 500 N with a frequency of 1 Hz. Moreover, the relationship between the output voltage and the load resistance is displayed in Figure S3. The power density increased as load resistance increased from 1 to 0.2 MΩ, and then decreased as load resistance further increased to 1 GΩ. The maximum power density was 18.4 W/m^2^ at the optimum resistance of 0.2 MΩ, which is efficient to power micro-nano devices. Furthermore, the as-obtained MCM-TENG was used to charge commercial capacitors of 1 μF, 2.2 μF, 4.7 μF, and 10 μF. The charging curves are displayed in Figure 5b. The capacitor of 1 μF can be charged to 3 V in 200 s. Apparently, the charging rate was inversely proportional to the capacitance. In addition, the stability test was conducted, as shown in Figure 5c. The MCM-TENG was measured under a force of 50 N and a frequency of 2 Hz. It can be seen that the output voltage was stable after about 2000 cycles. It demonstrated a long service time, which is important for practical applications.

**Figure 6:**
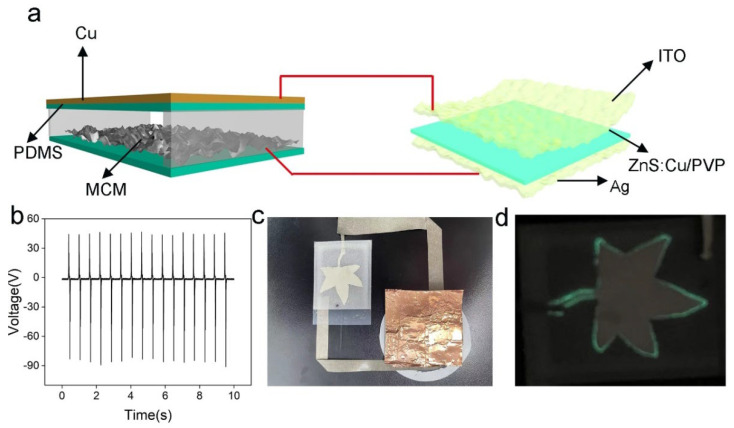
A self-powered display system driven by the MCM-TENG. (**a**) A schematic illustration of the self-powered display system. (**b**) The output voltage of MCM-TENG. (**c**) The photograph of a self-powered display system. (**d**) The display pattern driven by MCM-TENG.

Due to the intrinsic high AC output voltage, the TENG has advantages in powering flexible ACEL devices. Thus, it is promising to integrate TENG and ACEL to realize a self-powered display system. Figure 6a schematically illustrates the structure of a self-powered display system. The ZnS:Cu/PVP was the emission layer, while the ITO and Ag film were the two electrodes. The Cu and MCM electrodes of TENG were connected to the two electrodes of ACEL. Moreover, the ACEL was patterned as a leaf structure. The TENG with a diameter of 7 cm was pressed by the palm, and the output voltage is shown in Figure 6b. The output voltage was about 90 V at a frequency of 2 Hz, which was efficient enough to power ACEL. Furthermore, the photograph of the self-powered display system is shown in Figure 6c. The two electrodes were connected by a conductive Ag tape. By intermittently patting the MCM-TENG, the ACEL was brighter and darker at times. The photograph of ACEL lighted by the TENG is shown in Figure 6d. In addition, the self-powered display system could be mounted on the skin surface to realize the human–machine interface application.

## 4. Conclusions

In summary, a performance-enhanced MCM-TENG was reported for powering flexible patterned ACEL devices. The MCM multilayer composite films were self-assembled through layer-by-layer vacuum filtration. It concurrently acted as a triboelectric layer and electrode layer due to its high conductivity and strength. Furthermore, various flexible TENGs based on pure MXene film, MC film, and MCM film were constructed to evaluate the effect of CNF concentration and number of layers on the output performance of TENG. It was demonstrated that the MCM-TENG realized the optimum output performance with an output voltage of 25.0 V, an output current of 0.95 μA, and transferred charges of 9.0 nC. Finally, a flexible self-powered display device was demonstrated by integrating the flexible TENG and ACEL. The MCM-TENG with an output voltage of ≈90 V at a frequency of 2 Hz was found to be efficient enough to power an ACEL device. Consequently, the flexible TENG demonstrates a promising potential in terms of self-powered displays and human–machine interaction.

## Data Availability

The data presented in this study are available on request from the corresponding author.

## Supplementary Materials

The following supporting information can be downloaded at https://www.mdpi.com/article/10.3390/ma15196770/s1, Figure S1: The element distribution of MCM thin film. (a) The C element distribution. (b) The Ti element distribution. Figure S2: The switching polarity test of the transferred charges and output current of the TENG. (a,b) Transferred charges and (c,d) Output current when forward-connected (left) and reverse-connected (right) to measument system. Figure S3: The relationship between the output voltage and load resistances.
