# Supplementary material for: A Flexible Triboelectric Nanogenerator Based on Multilayer MXene/Cellulose Nanofibril Composite Film for Patterned Electroluminescence Display
Source: Materials (Basel). 2022 Sep 29;15(19):6770. doi: 10.3390/ma15196770 (PMC9571373; doi:10.3390/ma15196770)
Supplement: Supplementary file 1 [file materials-15-06770-s001.zip › materials-1906175-supplementary.pdf]

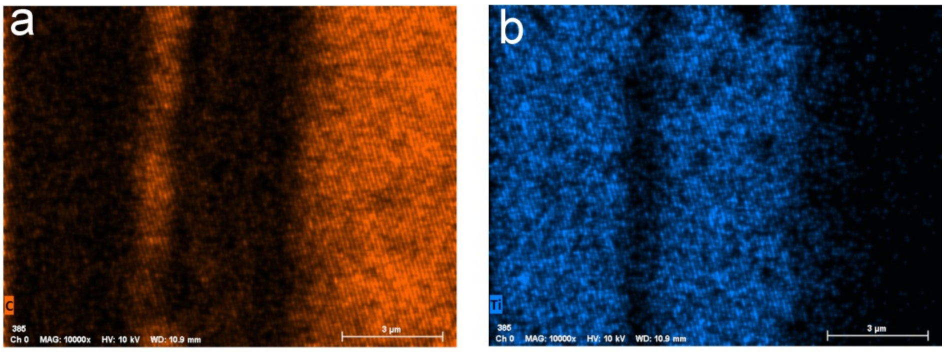

Figure S1. The element distribution of MCM thin film. (a) The C element distribution. (b) The Ti element distribution.

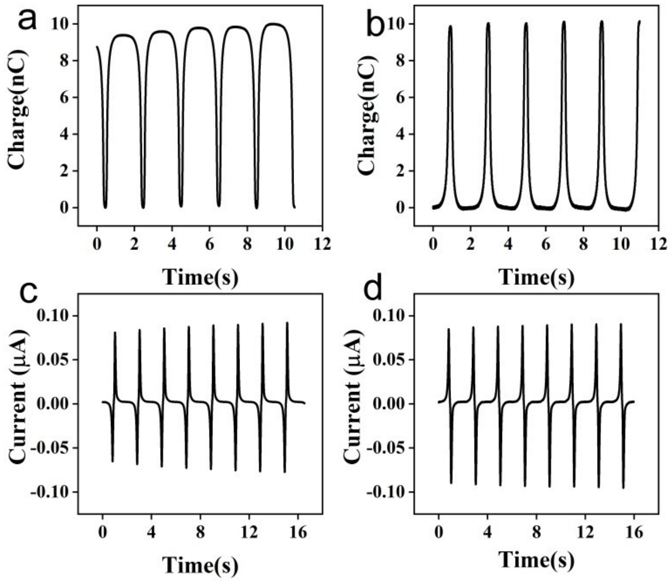

Figure S2. The switching polarity test of the transferred charges and output current of the TENG. (a-b) Transferred charges and (c-d) Output current when forward-connected (left) and reverse-connected (right) to measument system.

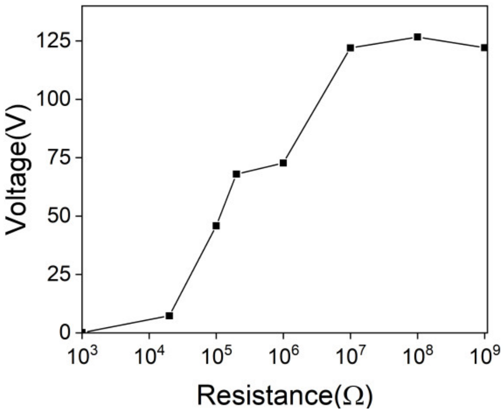

Figure S3. The relationship between the output voltage and load resistances.
